# Identification of male and female pupal characteristics of *Zeugodacus cucurbitae* (Coquillett) via machine vision

**DOI:** 10.1371/journal.pone.0264227

**Published:** 2022-03-24

**Authors:** Yuyang Lian, Aqiang Wang, Bei Zeng, Heming Yang, Jinlei Li, Sihua Peng, Shihao Zhou

**Affiliations:** Sanya Nanfan Research Institute of Hainan University, Hainan, Sanya, China; Chinese Academy of Agricultural Sciences Institute of Plant Protection, CHINA

## Abstract

Images of original pupae of *Zeugodacus cucurbitae* (Coquillett) were normalized, grayed, and segmented to identify male and female pupae of this species via machine vision. The image of each pupa was divided into 25 small areas. The differences in surface texture features in each small area within 11 days were compared. The texture characteristics of both male and female pupae were screened by combining the eclosion of both sexes of *Z*. *cucurbitae* (Coquillett). Results indicated that the pectinate setae on the abdominal backplane could be used as a basis for the identification of the male and female pupa of *Z*. *cucurbitae* (Coquillett). Moreover, machine vision correctly identified these characteristics with an accuracy of 96.0%. This study lays a foundation for the identification of male and female pupae using machine vision and also for the comprehensive control of *Z*. *cucurbitae* (Coquillett).

## 1 Introduction

*Zeugodacus cucurbitae* (Coquillett) (Diptera: Tephritidae) is an economically important insect pest of fruits and vegetables, with multiple hosts and a wide distribution. Adults of *Z*. *cucurbitae* (Coquillett) fly flexibly and do not feed on fruits and leaves. Thus, the traditional method of spraying crops with insecticides is unsuitable for their control. As an environmentally friendly pest control method, the sterile insect technique (SIT) has been successfully applied for the control of various pests because it is species-specific, eco-friendly, and can be applied to large areas [1]. The main strategy of SIT involves artificially raising a large number of target pests. After radiation treatment, sterile male individuals are continuously released to mate with wild female targets to produce sterile offspring, thereby controlling the population of the target pests below the economic threshold and even completely eradicating them [2]. Researchers in Hawai’i and Australia were the first to release sterile males of *Z*. *cucurbitae* (Coquillett) to eradicate this pest from the Northern Mariana Islands [3]. Some studies have demonstrated that releasing sterile male insects is more effective than releasing bisexual sterile insects. Thus, females insects must be removed before sterile male insects are released [4–6]. Thus, a technology that can rapidly and efficiently identify the sex of insects must be developed.

Morphological differences between the sexes of insects are largely used to distinguish them. For example, the male and female pupae of *Aedes* and *Culex* (Diptera) exhibit sexual dimorphism in terms of size, which is used for their manual separation [7]. Also, primers of molecular markers based on different sequences of the sex pheromone-binding protein gene of *Spodoptera frugiperda*, can be quickly and effectively used to identify their sexes at different developmental periods [8]. Pan et al. were able to identify accurately (100.0%) the sexes of adult *Histia rhodope* Cramer individuals by analyzing the left and right forewings using an image processing technology [9]. For *Z*. *cucurbitae* (Coquillett) differences in the external genitalia at the adult stage are the main characteristics used to distinguish the male and female [10]. Sex separation at the adult stage is complicated, and can easily cause mechanical damage to the insect body or affect its activity. A previous study showed that removal of females at the early developmental stages, was beneficial to avoid competition between sexes [11]. In many holometabolous insects, such as *Bactrocera dorsalis* [12] and *Aedes albopictus* [13], sex separation is usually conducted at the pupal stage. With the improvement in computer processing speed, image recognition methods based on machine vision have become highly efficient and accurate; these methods have been applied for the sex identification of insect pests [14].

The machine vision technology is a non-contact measurement technology that uses an optical equipment to obtain real images and then analyzes them through image processing technology, to obtain the required information or controls mechanical actuators to complete preset operations [15]. For example, Zhang et al. used computer vision technology to classify the male and female pupae of *Helicoverpa assulta* and *Helicoverpa armigera* effectively; the accuracy of recognition reached 87.5% and 82.5%, respectively [16]. In this study, the feasibility of the use of the machine vision technology for the identification of the characteristics of the male and female pupae of *Z*. *cucurbitae* (Coquillett) was evaluated. This study provides technical support to the prevention and control of this pest.

## 2 Materials and methods

### 2.1 Insect source management

The insects were collected from balsam pear fields (109°29ʹE, 19°30ʹN) near Nada Town, Danzhou City, Hainan Province, China. A population was raised on artificial diet in the laboratory. The larval diet contained 100 g yeast powder, 500 g corn flour, 4 g sodium benzoate, 100 g sucrose, 500 g pumpkin, 4 mL concentrated hydrochloric acid, and 500 mL water. The adult diet contained yeast powder and sucrose at a ratio of 1:1 (W:W). A stable temperature-sensitive laboratory population was established. The average indoor temperature was 25 C±1°C, and the other conditions were 70%±5% RH and 14:10 h of light and dark cycles.

### 2.2 Test reagents and materials

The reagents used in this study, such as yeast powder, corn flour, sodium benzoate, concentrated hydrochloric acid, and sucrose, were all purchased from Hainan Qingfeng Biotechnology Co., Ltd (China). The pumpkins were purchased from local markets.

### 2.3 Pupa collection and image acquisition

500 newly-pupated pupae were randomly selected and placed in custom-made square boxes, with one pupa in each grid. Images were continuously captured using the Nikon SMZ800N microscope and ImageView X64 edition at a fixed time every day until the pupae emerged, which were then treated with water for 1.5 h on the 8^th^ day.

### 2.4 Image pre-processing

Pupal images were pre-processed and then their features were compared to facilitate image variance analysis. The main processes included image pixel range normalization, color channel processing and conversion, pupal region extraction, calculation of the minimum enclosing rectangle of the region, image conversion to normal, clipping, size normalization, and grid differentiation according to the angle of the enclosing rectangle. Finally, each small grid region after division was compared, and differences were judged by comparing the texture information and gray features in the region.

#### 2.4.1 Image range normalization

The collected images were three-channel color images in tif format, and the pixel range was [0, 65520]. The range was normalized to [0, 255] to facilitate image processing.

#### 2.4.2 Color image space conversion

The collected images were color images, and they were directly transformed into grayscale images with low contrast and more surrounding interference, which were not conducive to the extraction of the pupal area. Therefore, the RGB space of the images was converted into HSV color space (H hue, S saturation, and V brightness). According to the analysis, the contrast of the whole region in the S channel, namely, color saturation, was the best and thus the most convenient for pupal segmentation.

#### 2.4.3 Region segmentation of pupae

The bright color area from the S-channel image was extracted using the Ostu algorithm. After extracting the area, the holes were filled with internal filling, and the surrounding area was corroded by morphological opening operation to obtain the pupal area.

#### 2.4.4 Calculation of the minimum enclosing rectangle of pupae

The minimum enclosing ellipse was first calculated for the region, and then the minimum enclosing rectangle was calculated on the basis of the ellipse region to improve the accuracy of rectangle calculation. According to the calculated angle, the image was straightened.

#### 2.4.5 Image clipping and normalization

Using the desired area, the pupal part was cut from the image, and the image size was normalized to a width of 2800 pixels and height of 1300 pixels to facilitate meshing and division of the images into equal parts.

#### 2.4.6 Image meshing and division into equal parts

The cropped small pictures were equally divided into 25 small areas, and changes in the texture and color in each area of the 11 pictures were compared.

## 3 Results and analysis

### 3.1 Mesh analysis of pupal images

**Fig 1:**
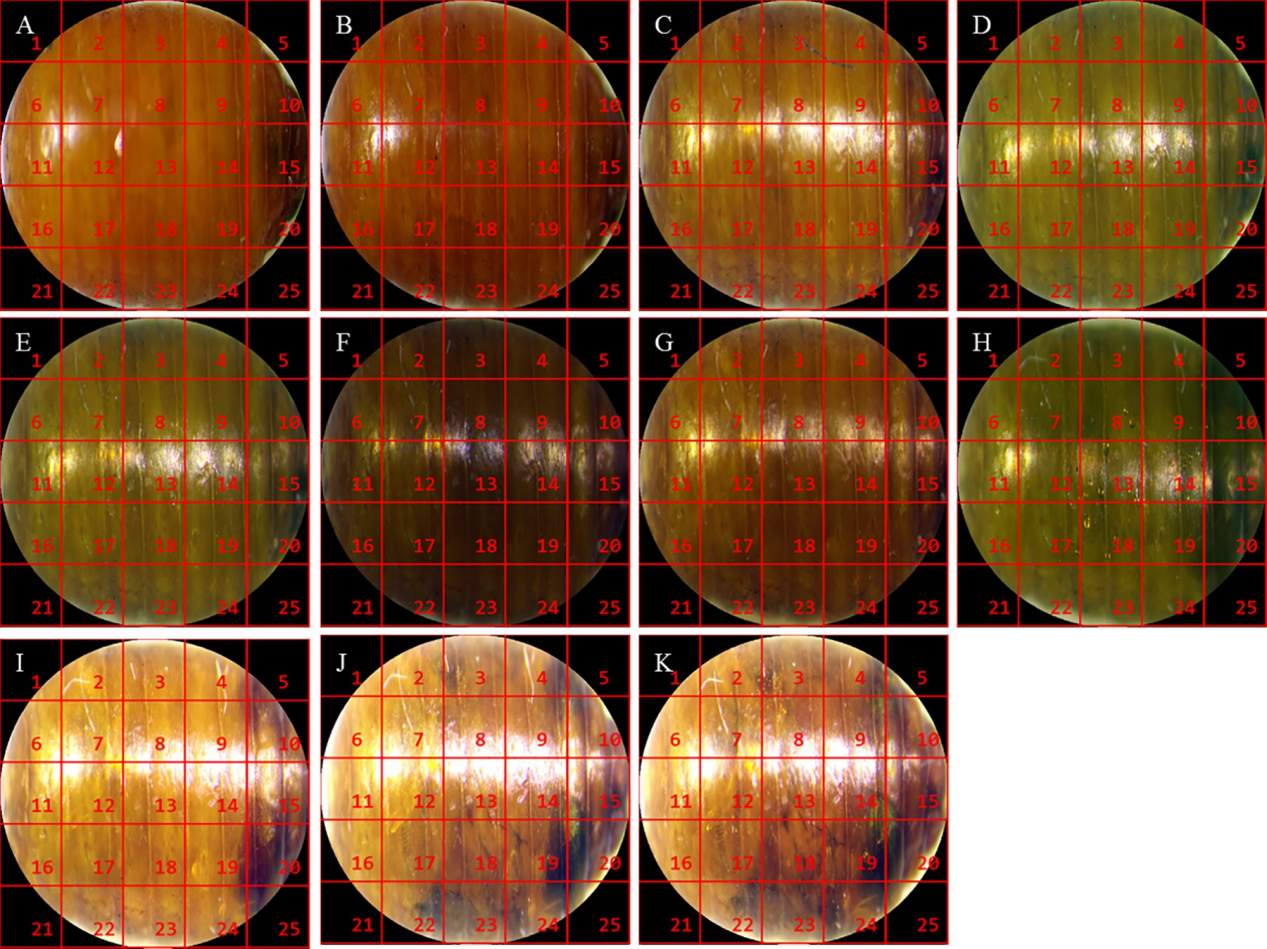
Meshed and equalized pupal images. Note: Fig 1A–1K respectively represent the mesh characteristics of *Z*. *cucurbitae* (Coquillett) pupae from day 1 to day 11.

**Fig 2:**
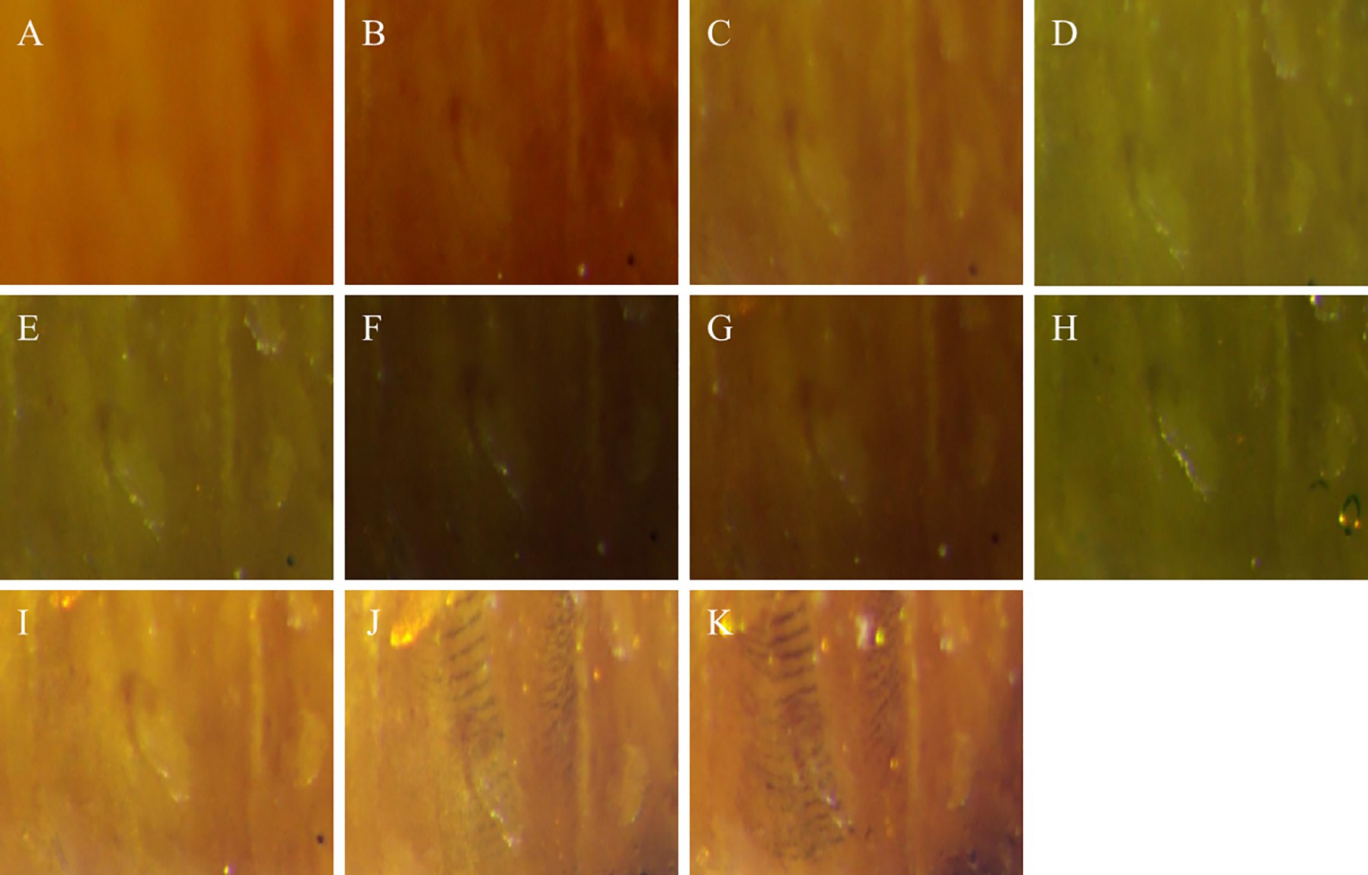
Enlarged view of area 17 of pupal image. Note: Fig 2A–2K respectively represent the changes of pupae characteristics from day 1 to day 11 in grid 17.

**Fig 3:**
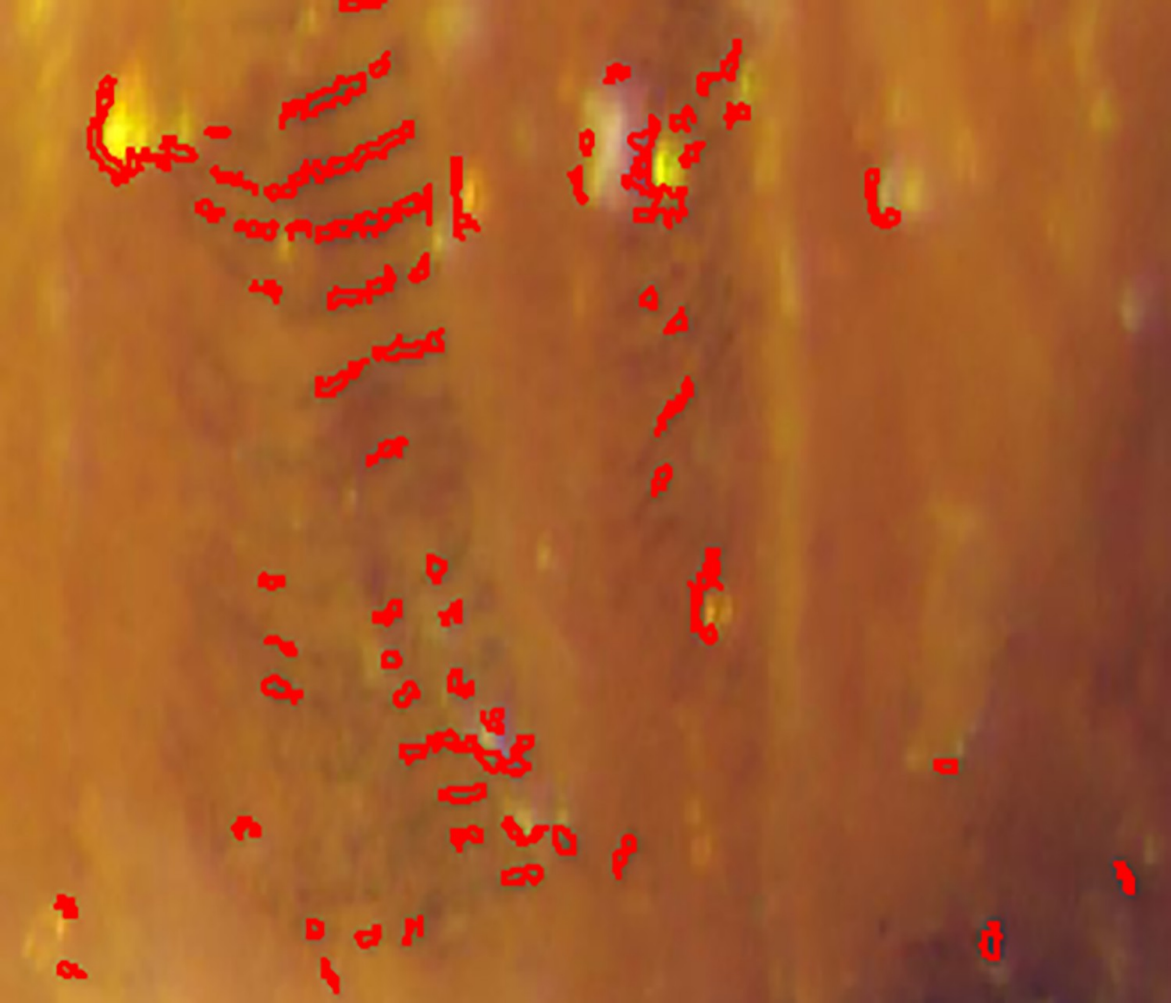
Characteristics of male and female pupae in area 17.

After the meshed pupal images were divided into equal parts (Fig 1), the texture characteristics of each region were analyzed. The texture characteristics of pupal surfaces in the 17th grid substantially changed over time. Partial enlargement of this grid showed dark stripes on the 10- and 11- days old pupae (Fig 2). This feature was then used to determine the sex of the pupae (Fig 3).

### 3.2 Identification of the characteristics of male and female pupae of Z. cucurbitae (Coquillett)

**Fig 4:**
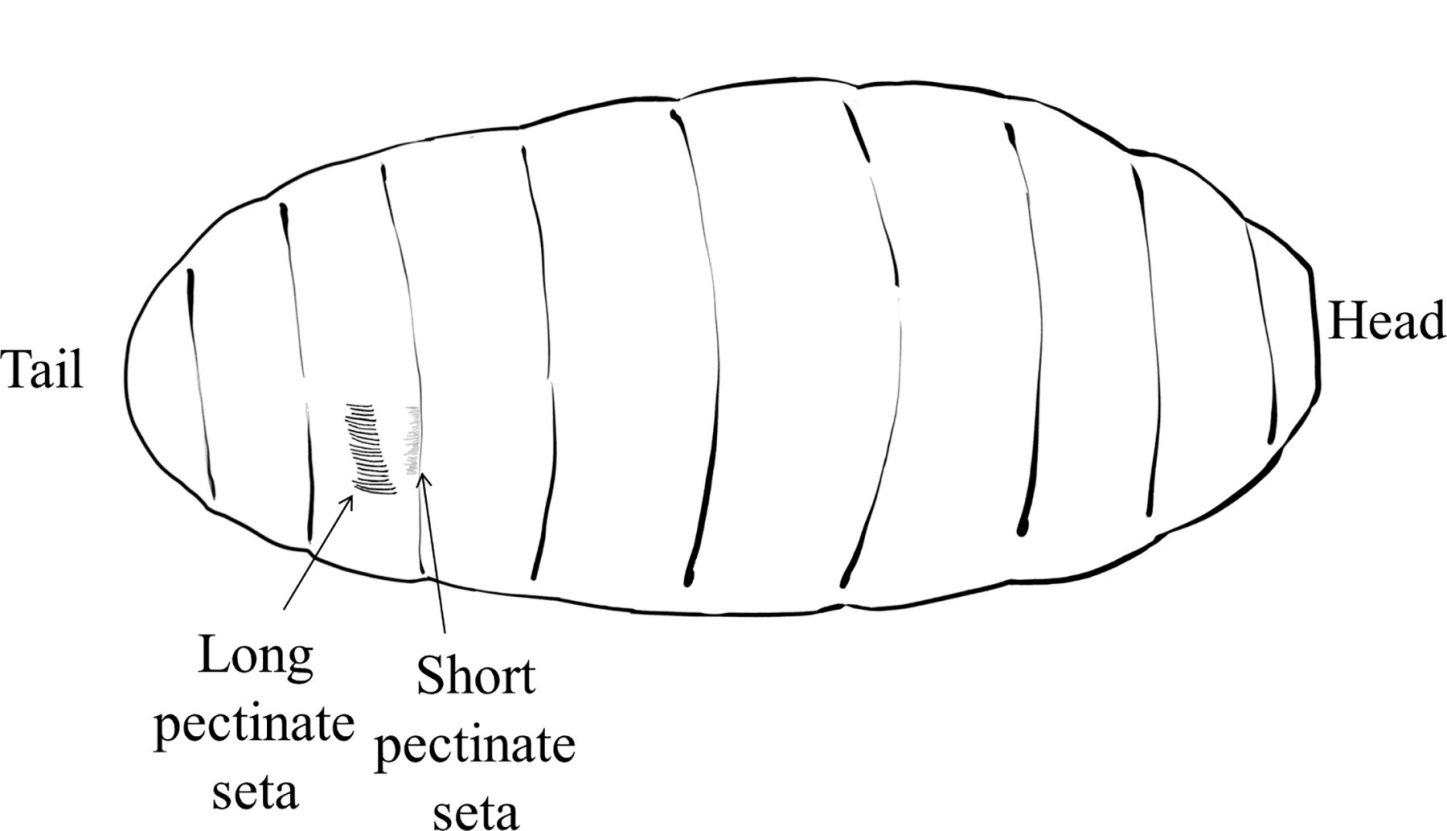
Side view of pupa.

Based on the results of the analysis of position and length differences, the dark streaks in region 17 were divided into two parts: one part was the long and dark pectinate seta in the middle of the third segment of the pupal shell tail, and the other part was the short and light pectinate seta at the junction of the third and fourth segments of the pupal shell tail. The length of the pectinate setae was also different owing to developmental differences among *Z*. *cucurbitae* (Coquillett)individuals. In this study, the longest and shortest lengths of the longer pectinate setae were 0.18 and 0.05 mm, respectively. The longest and the shortest lengths of the shorter pectinate setae were 0.05 and 0.02 mm, respectively. The longer pectinate setae were identified in males only, whereas the shorter pectinate setae were identified in both sexes. Therefore, the differentiation between male and female pupae, were based on the longer pectinate setae. About 16–20 pectinate setae were observed in the penultimate segment of the posterior margin (tail) of male pupae (Fig 4), which appeared 2 days before eclosion.

### 3.3 Verification of eclosion results

Machine vision analysis recognized pectinate setae in the pupal shell of 124 pupae, but this character was not recognized in the 376 remaining pupae. Pupal images were continuously taken for 11 days (feathering on the 12th day). All the nine pupae with pectinate setae were males. Among the 376 pupae without pectinate seta, 18 feathered into males, 310 into females and 48 did not feather (Table 1). The recognition accuracy was 96.0%.

**Table 1:** Identification results of male and female pupae.

The type of pupae	Number of pupae observed	Number of adult emergence			Accuracy of sex discrimination /%
		female	Male	unfledged	
No pectinate seta	376	310	18	48	94.5%
pectinate seta	124	0	124	0	100.0%

## 4 Discussion

The machine vision technology uses image sensors instead of human eyes to obtain object images and converts them into digital formats. A computer is then used to simulate human discrimination criteria to analyze and identify these images [17]. The machine vision system for identifying insects is divided into three consecutive stages: image capture, feature extraction and classification. Image capture refers to the photographs of insects; feature extraction involves obtaining useful visual information from pictures, and classification entails performing operations using the extracted information [18]. In this study, a stereomicroscope was connected to a computer imaging system for image capture. The pectinate setae of male pupae of *Z*. *cucurbitae* (Coquillett) were present on the abdominal backplane. Before image capture, the pupae were fixed with tapes (while avoiding stomata) to obtain the best classification view. Image segmentation is a key step in feature extraction, and its accuracy and quality directly affect the recognition and classification of insects [19]. The influence of the surrounding environment on the pupal area was eliminated and the subsequent image segmentation was facilitated by converting the RGB space of the image into the HSV color space. Afterward, the S-channel was extracted, and the optimal threshold was calculated using the Ostu algorithm to separate the foreground pixel from the background pixel. The Ostu algorithm can quickly binarize images [20]. For example, Zhu and Zhang set specific thresholds to binarize the wing images of lepidopteran insects, which segmented the foreground region from the background to enable their accurate identifications [21]. In the present study, hole filling and morphological analysis of the meshed segmented pupal images were conducted. After comparison, the pectinate setae were used as the feature to distinguish male and female pupae of *Z*. *cucurbitae* (Coquillett). Machine vision technology was then applied for the identification of this distinguishing feature.

All the 124 pupae with pectinate setae were males. Among the 328 pupae that survived and did not have pectinate setae, 18 were males and 310 were females, indicating that this technology had a high identification accuracy for males but a relatively low identification accuracy for females (94.5%). Thus, the presence of the pectinate setae can only be used to identify male pupae of *Z*. *cucurbitae* (Coquillett). However, some male pupae had no pectinate setae probably because of large differences in their ontogenies and the dysplasia of some male pupae. Studies have shown that pupae of *Z*. *cucurbitae* (Coquillett) have many air pockets between the inner layer and the hardened wall, thereby making them opaque. Placing the pupae in water for 1–2 h would cause the water to enter the air pockets, to make the pupae transparent and this operation has no obvious side effects on specimen observation [22]. On the 8^th^ day, 500 pupae were treated with water for 1.5 h, but no pectinate setae were identified in the images on the 8^th^ and 9^th^ days. The pectinate setae of the nine male pupae were identified on the 10^th^ day likely because the color of the early pectinate setae was light and thus could not be detected in the images. The pectinate setae were recognized only 2 days before eclosion.

The machine vision technology can provide technical support for the accurate identification of male and female pupae of *Z*. *cucurbitae* (Coquillett), This information can be used to monitor and control this pest effectively. The male sterility technology is an important means of preventing and controlling the reproduction of *Z*. *cucurbitae* (Coquillett). It involves artificially breeding a large population of the pest. Males are subjected to radiation treatment, making them sterile. These individuals are then released into the field [23], and sterile eggs are produced after they have mated with wild females [24]. After mating with sterile males, the ability of wild female to mate repeatedly is inhibited, thereby greatly reducing the number of eggs that they can lay [25]. This ensures the safe and efficient control of *Z*. *cucurbitae* (Coquillett). Collection of males for sterility treatment requires a lot of time and manpower. Therefore, the combination of the machine vision technology with the male sterility technology, to single out male pupae can substantially reduce the workload involved. Li et al. used the RNAi technology to interfere with the *lnC94638* gene expression in *Z*. *cucurbitae* (Coquillett) males to reduce their sperm number and fertility [26]. Moreover, they used the same technology to interfere with the *ZcVMP26Ab* gene in females to achieve low egg hatchability [27]. Given that many of the target genes in RNAi technology are male- or female-specific genes, *Z*. *cucurbitae* (Coquillett) males and females must be separated prior to the conduct of experiments. The use of machine vision in analyzing and identifying the male and female pupae of *Z*. *cucurbitae* (Coquillett) can simplify the experimental steps, for a quick and accurate determination of their sexes.

As a new interdisciplinary subject, the application of machine vision technology for insect recognition and pest control started late; nevertheless, research on these topics using this technology is rapidly advancing [28]. For example, Gong and Li adopted a computer digital image processing technology and a neural network technology to identify automatically the sexes of silkworm pupae of two varieties, with an accuracy of 98.0% and 96.0% for each [29]. In this study, the accuracy of identifying the male and female pupae of *Z*. *cucurbitae* (Coquillett) reached 96.0%. If the female characteristics can be defined, then the recognition accuracy can be improved further. In this study, five specimens were unfledged pupae because they died, but the dead pupae could not be detected during the image recognition process. However, Ahmad et al. used RGB images with specific color wavelengths for each pixel to distinguish the living and dead pupae of the bagworm *Metisa plana* Walker (Lepidoptera: Psychidae) [30]. Combined with the machine vision technology, this method can identify dead pupae before emergence and promptly remove them, thereby improving the accuracy of the identification of male and female pupae. This study provided a reference for the development of effective control strategies for *Z*. *cucurbitae* (Coquillett) on the basis of the identification of sexes of this species.
